# Numerical and Experiment Analysis of Sapphire Sandwich-Structure Fabry–Perot Pressure Sensor through Fast Fourier Transform and Mean Square Error Demodulation Algorithm

**DOI:** 10.3390/ma17153649

**Published:** 2024-07-24

**Authors:** Zhenyin Hai, Zhixuan Su, Rui Liang, Maocheng Guo, Hongtian Zhu, Jun Chen, Qi Zhang, Yue Chen, Runze Lin, Yihang Zhang, Zewang Zhang, Chenyang Xue

**Affiliations:** 1 School of Aerospace Engineering, Xiamen University, Xiamen 361005, China; suzhixuan@stu.xmu.edu.cn (Z.S.); 19920221151622@stu.xmu.edu.cn (M.G.); 35020230156555@stu.xmu.edu.cn (H.Z.); 19920231151668@stu.xmu.edu.cn (Q.Z.); 35120231151728@stu.xmu.edu.cn (Y.C.); 34520202201658@stu.xmu.edu.cn (R.L.); 15259300843@163.com (Y.Z.); xuechenyang@nuc.edu.cn (C.X.); 2 School of Instrument and Electronics, North University of China, Taiyuan 030051, China; s202206095@st.nuc.edu.cn; 3 School of Electronic Engineering, Ocean University of China, Qingdao 266000, China; 18335160365@163.com; 4 School of Opto-Electronic and Communication Engineering, Xiamen University of Technology, Xiamen 361005, China

**Keywords:** mean square error, Fast Fourier Transform, demodulation algorithm, sapphire, Fabry–Perot, pressure sensor

## Abstract

Pressure sensors prepared from sapphire exhibit excellent characteristics, including high-temperature resistance, high hardness, and resistance to electromagnetic interference. A Fast Fourier Transform and Mean Square Error (FFT-MSE) demodulation algorithm was employed to demodulate a sapphire sandwich-structure Fabry–Perot (F-P) pressure sensor. Through simulation analysis, the experimental results indicated that the demodulation error of the air cavity length in the range of 206 μm to 216 μm was less than 0.0008%. Compared to single demodulation methods and combined demodulation methods based on FFT or Minimum Mean Square Error (MMSE), the method proposed in this work reduced the demodulation error by more than three times and increased accuracy by more than six times. The algorithm was utilized to demodulate the sapphire sandwich-structure F-P pressure sensor, and the test results indicated that the fitting error of the sensor was less than 0.025% within the pressure range of 0 MPa to 10 MPa. The repeatability error was less than 0.066%, the zero-point deviation was 1.26%, and the maximum stability deviation was 0.0063% per 30 min. The algorithm effectively demodulated the actual cavity length variation in the sapphire sandwich-structure F-P pressure sensor, providing a solution for the performance evaluation of the sapphire sandwich-structure F-P pressure sensor.

## 1. Introduction

The measurement of pressure values in the operating states of key components in high-hypervelocity vehicles and advanced aerospace engines is essential for assessing their operating states, structural optimization, and safety performance [1]. For example, during aero-engine testing, pressure measurements are required for intakes, combustion chambers, tail nozzles, and exhaust outlets [2,3,4]. The melting point of sapphire material is as high as 2040 °C [5], and the pressure sensors made from it possess superior properties such as high-temperature resistance, high hardness, anti-electromagnetic interference, and high-temperature stability [6,7,8]. It is reported that pressure sensors based on sapphire material are mainly F-P pressure sensors [9,10,11,12,13,14]. Sapphire Fabry–Perot (F-P) pressure sensors have high accuracy, high stability, and high-temperature environment adaptability, making them more suitable for pressure measurement in extreme aerospace environments. The main demodulation algorithms for sapphire F-P pressure sensors include bimodal methods [15,16,17], Fourier transform methods [18,19,20], phase quadrature methods [21,22,23], and mutual correlation methods [24,25,26]. Zhiqiang Shao et al. achieved the demodulation of the sapphire F-P cavity length ranging from 103 µm to 283 µm by using the mutual correlation function method with a pressure range of 0 MPa to 5 MPa [27]. Zilong Guo et al. achieved the demodulation of the sapphire F-P cavity with a length range of 60 µm to 95 µm using the white light interference method with a pressure range of 0 MPa to 2.9 MPa and accuracy of up to 0.04% [28]. Yang Cui et al. achieved the demodulation of the quartz F-P cavity with a length range of 614 µm to 630 µm using the white light interference method with a pressure range of 0 MPa to 10 MPa [29].

This work combines the Fast Fourier Transform (FFT) and Mean Square Error (MSE) demodulation methods, interrelating the computational results to achieve air cavity length demodulation in a sapphire sandwich-structure F-P cavity. The objective was to optimize the low accuracy of FFT and the instability of MSE. Simulation results indicated that the maximum deviation is less than 0.1 nm, and demodulation accuracy reaches 0.0008% using the FFT-MSE demodulation method. During actual testing, the sapphire sandwich-structure pressure sensor undergoes multiple large-range pressure tests from 0–10 MPa. The fitting error of this sensor is less than 0.016%, the repeatability error is less than 0.066%, and no mode-jumping problem arises, demonstrating that the method has good reliability and stability. This method provides a reliable solution for determining the air cavity length of sapphire sandwich-structure F-P cavity.

## 2. Test Systems and Sensor Structures

### 2.1. Test System

**Figure 1:**
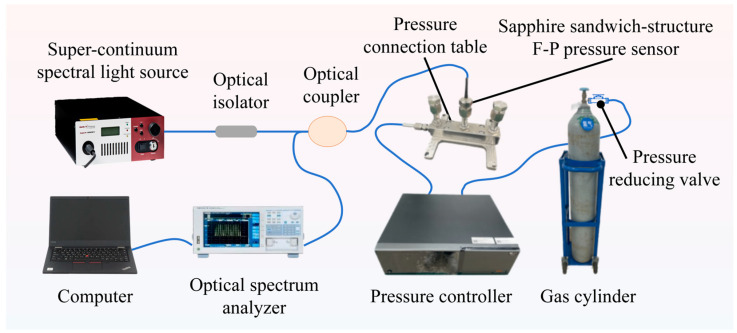
Diagram of the sapphire sandwich-structure F-P pressure sensor test system.

Figure 1 illustrates the sapphire sandwich-structure F-P pressure sensor test system, which comprises a optical spectrum analyzer (DAQ6370, Yokogawa Measurement Technology Co., Ltd., Tokyo, Japan), a super-continuum spectral light source (SuperK COMPACT, NKT Photonics A/S, Copenhagen, Denmark), a pressure controller (860-25M, Beijing ConST Instruments Technology Inc., Beijing, China), an optical isolator, an optical coupler, a computer, a pressure connection table, a gas cylinder, and a pressure reducing valve. The super-continuum spectral light source emits laser light with a wavelength of 400 nm to 2400 nm. Figure S1 illustrates the coupling efficiency between the signal emitted by the super-continuum spectral light source and the single-mode fiber, which ranges from 17.71% to 23.8%, within the wavelength range of 1520 nm to 1570 nm. The laser light passes through the optical isolator and optical coupler before entering the sapphire sandwich-structure F-P pressure sensor. Its reflection spectrum then passes through the optical coupler, and the optical spectrum analyzer collects the reflection spectrum signals (sampling wavelength range: 1520 nm to 1570 nm, number of sampling points: 1001, scanning speed: 1×). The collected reflectance spectral signals are transferred to a computer with demodulation system software written in MATLAB (version number: 9.6.0.1047502), which demodulates the changes in the air cavity length of the sapphire sandwich-structure F-P cavity. The pressure in the gas cylinder is approximately 15 MPa, reduced to about 12 MPa by a pressure reducing valve. The pressure controller connects the gas cylinder to the pressure connection table, regulating the pressure within it. The sapphire sandwich-structure F-P pressure sensor is mounted onto the pressure connection table.

### 2.2. Sensor Structure

**Figure 2:**
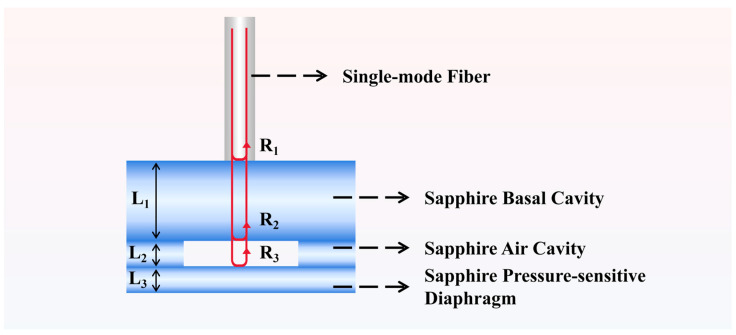
The sensitive core of the sapphire sandwich-structure F-P cavity.

Figure 2 illustrates the sensitive core of the sapphire sandwich-structure F-P cavity, which comprises a basal cavity, an air cavity, and a pressure-sensitive diaphragm, all constructed from sapphire. The optical fiber consists of quartz. The super-continuum spectral light source emits a beam that reflects off the upper surface of the basal cavity (reflectivity R_1_), the lower surface of the basal cavity (reflectivity R_2_), and the upper surface of the pressure-sensitive diaphragm (reflectivity R_3_), resulting in an interferometric reflectance spectrum. The sapphire basal cavity diaphragm thickness (d1) is 600 μm, with a diameter of 8 mm. The sapphire pressure-sensing diaphragm thickness (d3) is 200 μm, with a diameter of 8 mm. The sapphire air cavity diaphragm thickness (d2) is 216 μm, with an outer diameter of 8 mm and an inner diameter of 4 mm. The quartz optical fiber is a single-mode fiber with a diameter of 125 μm.

**Figure 3:**
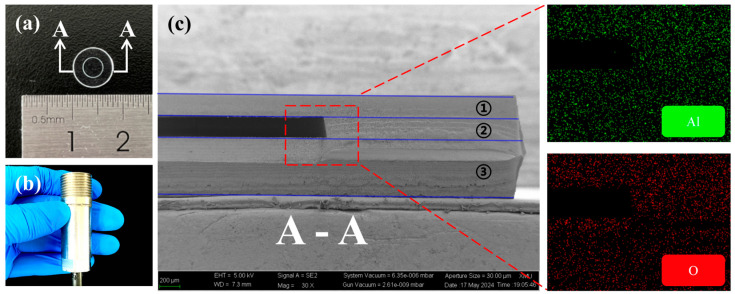
(**a**) The upper structure of the sensitive core of the sapphire sandwich-structure F-P pressure sensor. (**b**) Physical diagram of sapphire sandwich-structure F-P pressure sensor. (**c**) The micro-structure in the A-A cross section of the sensitive core of the sapphire sandwich-structure F-P pressure sensor and its corresponding surface element distribution (Al, O).

Figure 3a shows the upper structure of the sensitive core of the sapphire sandwich-structure F-P pressure sensor. The diameter of the sensitive core is approximately 8 mm. Figure 3b illustrates the physical diagram of the sapphire sandwich-structure F-P pressure sensor. The sensor is encased in 304 stainless steel and features a thread diameter of 20 mm with a pitch of 1.5 mm. The scanning electron microscope (SEM) manufactured by Carl Zeiss AG, model SUPRA55 SAPPHIRE, combined with energy-dispersive X-ray spectroscopy (EDS), is used to analyze the micro-structure and element distribution of the sensitive core of the sapphire sandwich-structure F-P pressure sensor. The micro-structure in A-A cross section of the sapphire sandwich-structure F-P cavity appears in Figure 3c, and the corresponding surface elements are Al and O. This is due to the main material composition of sapphire being Al_2_O_3_. From top to bottom, the layers are the sapphire pressure-sensitive diaphragm (①), sapphire air cavity diaphragm (②), and sapphire basal cavity diaphragm (③).

## 3. Simulation and Analysis

To effectively simulate the reflectance spectrum of the sapphire sandwich-structure F-P cavity, it is necessary to construct the reflectance spectrum using basal cavity reflectance spectrum expression, air cavity reflectance spectrum expression, and interferometric reflectance spectrum expression for both the basal and air cavities [28,30,31]. The basal cavity reflectance spectrum expression is as follows:

(1) $$I_{1}=2\sqrt{R_{1}R_{2}}cos(\frac{4\pi n_{1}L_{1}}{c}v)I_{0}$$

n_1_ denotes the refractive index of the basal cavity, L_1_ represents the basal cavity length, I_0_ indicates the incident spectrum, I_1_ denotes the basal cavity reflectance spectrum, c represents the speed of light, and v indicates the incident light frequency. The air cavity reflectance spectrum expression is as follows:

(2) $$I_{2}=2\sqrt{R_{2}R_{3}}cos(\frac{4\pi n_{2}L_{2}}{c}v)I_{0}$$

n_2_ denotes the refractive index of the air cavity, L_2_ represents the air cavity length, and I_2_ indicates the air cavity reflectance spectrum. The interferometric reflectance spectrum expression for the basal and air cavities is as follows:

(3) $$I_{3}=2\sqrt{R_{1}R_{3}}cos[2(\frac{4\pi n_{1}L_{1}}{c}v+\frac{4\pi n_{2}L_{2}}{c}v)]I_{0}$$

The reflectance spectrum expression for the sapphire sandwich-structure F-P cavity is as follows:

(4) $$I_{r}=(R_{1}+R_{2}+R_{3}+I_{1}+I_{2}+I_{3})I_{0}$$

**Figure 4:**
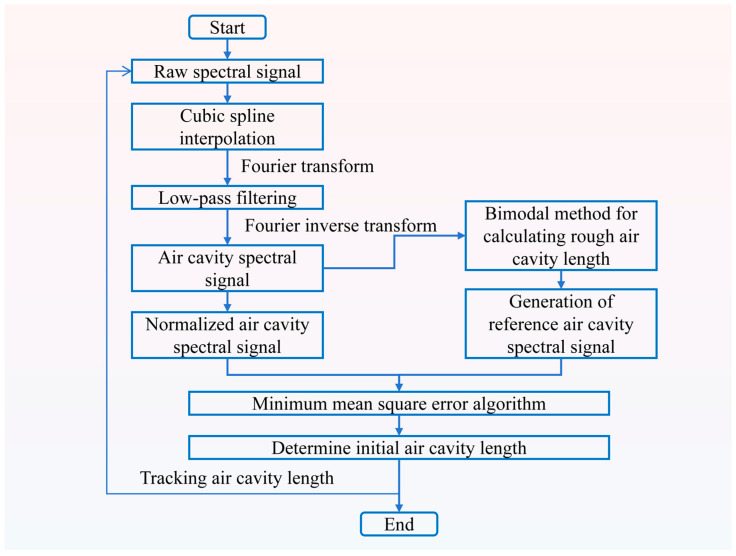
Flowchart of FFT-MSE demodulation algorithm.

**Figure 5:**
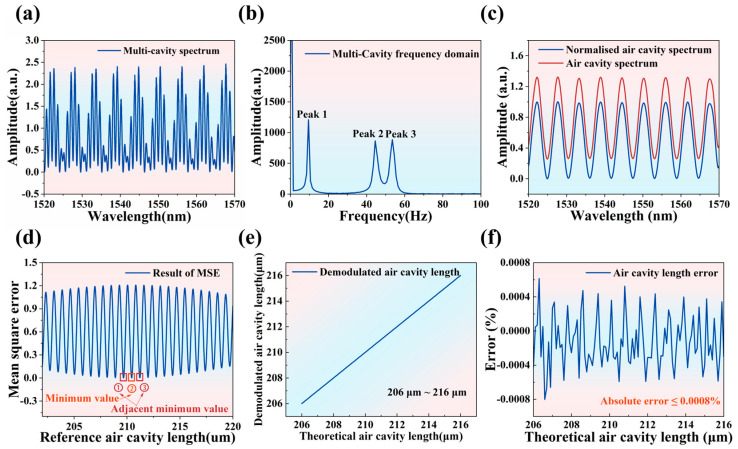
(**a**) Sapphire sandwich-structure F-P cavity reflectance spectrum after adding noise. (**b**) Sapphire sandwich-structure F-P cavity frequency domain spectrum. (**c**) Air cavity spectrum and normalized air cavity spectrum. (**d**) Result of MSE. (**e**) Result of demodulated air cavity length and theoretical air cavity length. (**f**) Simulation error curve.

The flowchart of the FFT-MSE demodulation algorithm is shown in Figure 4. The reflectance spectrum of the sapphire sandwich-structure F-P cavity (Figure S2) is plotted using Equation (4), with a wavelength range from 1520 nm to 1570 nm. To simulate the reflectance spectrum of the sapphire sandwich-structure F-P cavity more efficiently, normal distribution noise is introduced. The reflectance spectrum with added normal distribution noise is shown in Figure 5a. The signal-to-noise ratio (SNR) distribution in the wavelength range from 1520 nm to 1570 nm is shown in Figure S3, with the SNR greater than 35.1. The sapphire sandwich-structure F-P cavity reflectance spectrum is subjected to cubic spline interpolation. The frequency domain spectrum of the sapphire sandwich-structure F-P cavity is obtained by using the FFT (shown in Figure 5b). The frequency corresponding to the first peak is the air cavity frequency, the second peak corresponds to the basal cavity frequency, and the third peak results from the combined effect of the air cavity and basal cavity. The frequency domain spectrum of the sapphire sandwich-structure F-P cavity is low-pass filtered with a cut-off frequency of 20 Hz. The air cavity reflection spectrum is obtained by Fourier inverse transform and is then normalized as shown in Figure 5c. The normalization calculation formula is as follows:

(5) $$I_{4}=\frac{I_{2}-S_{\mathrm{down}1}}{S_{up1}}$$

I_4_ denotes the normalized air cavity reflectance spectrum, S_down1_ represents the lower envelope of the air cavity reflectance spectrum, and S_up1_ indicates the upper envelope of the air cavity reflectance spectrum. The rough air cavity length is then calculated using the bimodal method, which employs the wavelengths ($\lambda_{1}$ and $\lambda_{2}$) corresponding to the two adjacent wave peaks of the air cavity spectrum signal to determine the rough air cavity length ($L_{4}$). The calculation formula is as follows [32]:

(6) $$L_{4}=\frac{\lambda_{1}\lambda_{2}}{2(\lambda_{2}-\lambda_{1})}$$

The calculated L_4_ is used to construct the reference air cavity length ($L_{5}$), which ranges from $L_{4}-∆L$ to $L_{4}+∆L$, with a step size (∆d) of 10 nm, and ∆L is 10,000 nm. L_5_ is then substituted into Equation (2) to construct the reference air cavity reflectance spectrum. The normalized reference air cavity reflectance spectrum (I_5_) calculated using Equation (5). I_4_ and I_5_ are substituted into the MSE calculation formula; the result is shown in Figure S4. The MSE calculation formula is as follows:

(7) $$\mathrm{MSE}=\frac{1}{N}\sum_{i=1}^{n}{\left(I_{5}-I_{4}\right)}^{2}$$

where N denotes the number of sampling points of the sapphire sandwich-structure F-P cavity reflectance spectrum after three iterations of spline interpolation, and n represents the sample points of L_5_.

The minimum value of MSE corresponds to the reference air cavity length, identified as the first air cavity length. As shown in Figure 5d, after calculating the second MSE, the next step is to determine which of the reference air cavity lengths corresponding to “①”, “②”, and “③” is closest to the first air cavity length and then use this value as the air cavity length. Each subsequent air cavity length calculation references the immediately preceding air cavity length. The results of the simulation calculations for the variation in air cavity length from 206 μm to 216 μm are shown in Figure 5e. The simulation error curve is shown in Figure 5f, with the simulation error being less than 0.0008%. As shown in Table 1, compared with the dual peak method [15], dual/mult wavelength method [33,34], phase demodulation method [26,28,35], MMSE method [36,37,38], and FFT method [18,19,39], the method proposed in this work achieves a demodulation error that is more than three times smaller and accuracy that is more than six times higher. The simulation parameters not explicitly stated above are listed in Table 2.

## 4. Experiments

**Figure 6:**
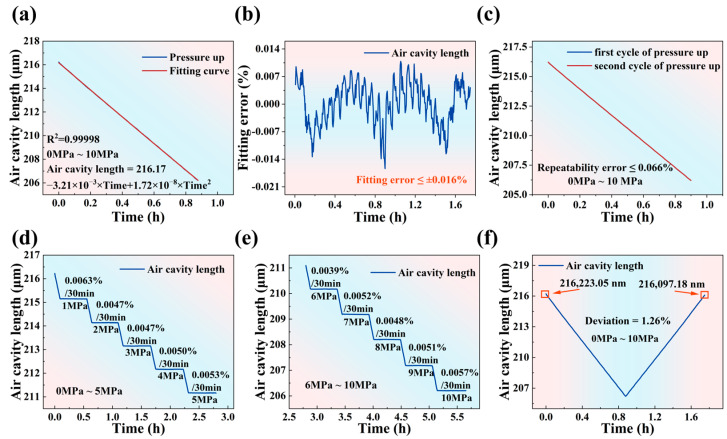
(**a**) Air cavity length fitting curve from 0 MPa to 10 MPa. (**b**) Air cavity length fitting error curve from 0 MPa to 10 MPa. (**c**) Air cavity length curve for two rounds of pressurization. (**d**) Stability at 1 MPa, 2 MPa, 3 MPa, 4 MPa, and 5 MPa. (**e**) Stability at 6 MPa, 7 MPa, 8 MPa, 9 MPa, and 10 MPa. (**f**) Boost and buck curves from 0 MPa to 10 MPa.

To effectively evaluate the feasibility of the demodulation algorithm, it is necessary to test the sapphire sandwich-structure F-P pressure sensor. As shown in Figure 6a, the sapphire sandwich-structure F-P pressure sensor is pressurized from 0 MPa to 10 MPa in a single round, with a boost rate of the pressure controller at 0.003 MPa/s. Second-order fitting equations are used for modeling, resulting in an R^2^ value of 0.99998. The fitting error curve is shown in Figure 6b, and the fitting error (e_f_) is calculated using the following formula:

(8) $$e_{f}=\frac{Y-Y_{f}}{Y_{F.S.}}\times 100\%$$

Y denotes the air cavity length curve, Y_f_ represents the fitting curve of the air cavity length, and Y_F.S._ indicates the variation range of the air cavity length. According to Equation (8), the absolute value of e_f_ is calculated to be less than 0.016%. To evaluate the repeatability of the sapphire sandwich-structure F-P pressure sensor, two rounds of 0 MPa to 10 MPa pressurization are performed, with the pressure controller’s boost rate at 0.003 MPa/s. The test results are shown in Figure 6c, and the repeatability error ($\delta_{R}$) is calculated as follows:

(9) $$\delta_{R}=\frac{∆Y}{Y_{F.S.}}\times 100\%$$

∆Y denotes the difference between the change in curves of the air cavity lengths of the two rounds, and Y_F.S._ represents the variation range of the air cavity length. According to Equation (9), the calculated $\delta_{R}$ is less than 0.066%, indicating that the sapphire sandwich-structure F-P pressure sensor has excellent repeatability. The stability of the sensor is a key indicator for evaluating its performance. Stability tests are conducted on the sapphire sandwich-structure F-P pressure sensor for 1 MPa to 10 MPa. The test results, shown in Figure 6d,e, indicate stability values of 0.0063%/30 min at 1 MPa, 0.0047%/30 min at 2 MPa, 0.0047%/30 min at 3 Mpa, 0.0050%/30 min at 4 MPa, 0.0053%/30 min at 5 MPa, 0.0039%/30 min at 6 MPa, 0.0052%/30 min at 7 MPa, 0.0048%/30 min at 8 MPa, 0.0051%/30 min at 9 MPa, and 0.0057%/30 min at 10 MPa. Notably, no “mode jumping” occurs during the 8 h continuous demodulation process. Figure 6f shows the 0 MPa to 10 MPa boost and buck curves, and the deviation ($\delta_{D}$) of the zero point is calculated as follows:

(10) $$\delta_{D}=\frac{∆Y{丨}_{P=0}}{Y_{F.S.}}\times 100\%$$

$∆Y{丨}_{P=0}$ the deviation value of the zero point, and Y_F.S._ indicates the variation range of the air cavity length. The pressure controller has a ramp-up and ramp-down rate of 0.003 MPa/s. After one round of ramp-up and ramp-down, the air cavity length offset of the sapphire sandwich-structure F-P pressure sensor at atmospheric pressure is 125.87 nm, and according to Equation (10), the $\delta_{D}$ is calculated to be 1.26%. The performance of the sapphire sandwich-structure F-P pressure sensor is accurately evaluated using the FFT-MSE algorithm, which provides a generalized method for calculating the cavity length of sandwich-structure F-P cavity.

## 5. Conclusions

In conclusion, through the simulation analysis of the sapphire sandwich-structure F-P cavity, experimental results show that the demodulation error of the air cavity length in the range of 206 μm to 216 μm is less than 0.0008%, indicating that the algorithm has excellent accuracy. Compared to single demodulation methods and combined demodulation methods based on FFT or MMSE, the method proposed in this work reduces demodulation errors by more than three times and increases accuracy by more than six times. Testing the application of the sapphire sandwich-structure F-P pressure sensor using the algorithm reveals a fitting error of less than 0.025%, a repeatability error of less than 0.066%, a deviation of the zero point of 1.26%, and a maximum stability deviation of 0.0063%/30 min in the pressure range of 0 MPa to 10 MPa. The test results show that the algorithm effectively demodulates the actual cavity length variation in the sapphire sandwich-structure F-P pressure sensor, providing a robust solution for its performance evaluation. The performance of the sapphire sandwich-structure F-P pressure sensor is accurately evaluated using the FFT-MSE algorithm.

## Figures and Tables

**Table 1:** Comparison of the simulation performance of different algorithms.

Main Method	Air Cavity Length (um)	Error (nm)	Error (%)	Refs.
PTP	2515–2610	<2000	2.11	[15]
DW	/	<35	/	[33]
EMW	/	<20	/	[34]
PCC	/	<0.3	/	[26]
WLNSC	60–95	<14	0.04	[28]
SCC	15–25	<0.43	0.0437	[35]
MMSE	16.03–16.14	<1.2	0.88	[36]
MMSE + nDFT	241–243	<0.5	0.4	[37]
MMSE + VR	/	<4.8	/	[38]
FFT	/	/	0.3	[18]
FFT + MLE	300.5–303.5	15	0.005	[19]
FFT + CZT	397–403	<0.6	0.01	[39]
FFT + MSE	206–216	<0.1	0.0008	This work

**Table 2:** Simulation parameters.

Parameters	Value
L_1_	600 μm
L_2_	206~216 μm
W	1520~1570 nm
I_0_	1 a.u.
R_1_, R_2_, R_3_	0.075
n_1_	1.765
n_2_	1.000
c	3 × 10^8^ m/s

## Data Availability

The original contributions presented in this study are included in the article/Supplementary Materials; further inquiries can be directed to the corresponding author.

## Supplementary Materials

The following supporting information can be downloaded at https://www.mdpi.com/article/10.3390/ma17153649/s1, Figure S1: The coupling efficiency results of the signal emitted by the super-continuum spectral light source and the single-mode fiber in the wavelength range of 1520 nm to 1570 nm; Figure S2: The reflectance spectrum of the sapphire sandwich-structure F-P structure; Figure S3: The signal-to-noise ratio (SNR) distribution in the wavelength range from 1520 nm to 1570 nm; Figure S4: Result of MSE.
