# Supplementary material for: Numerical and Experiment Analysis of Sapphire Sandwich-Structure Fabry–Perot Pressure Sensor through Fast Fourier Transform and Mean Square Error Demodulation Algorithm
Source: Materials (Basel). 2024 Jul 24;17(15):3649. doi: 10.3390/ma17153649 (PMC11313623; doi:10.3390/ma17153649)
Supplement: Supplementary file 1 [file materials-17-03649-s001.zip › materials-3096609-supplementary.pdf]

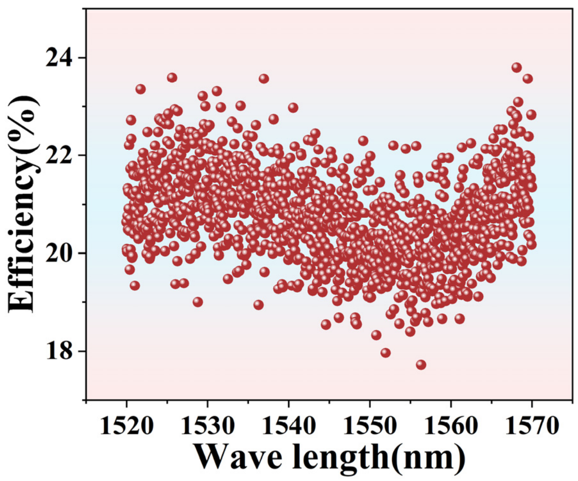

Figure S1: The coupling efficiency results of the signal emitted by the super-continuum spectral light source and the single-mode fiber in the wavelength range of 1520nm to 1570nm

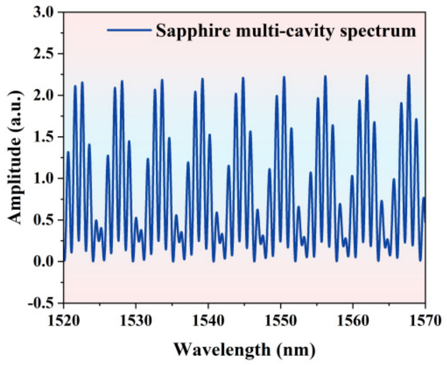

Figure S2: The reflectance spectrum of the sapphire sandwich-structure F-P structure.

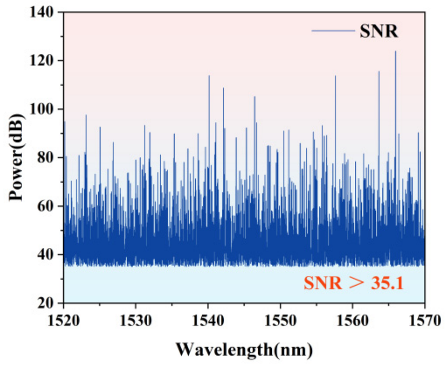

Figure S3: The signal-to-noise ratio (SNR) distribution in the wavelength range from 1520 nm to 1570 nm.

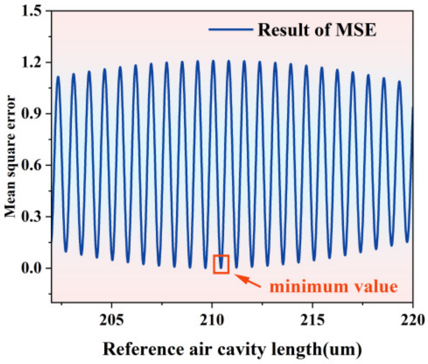

Figure S4: Result of MSE.
